# Regulatory Mechanisms and Promising Applications of Quorum Sensing-Inhibiting Agents in Control of Bacterial Biofilm Formation

**DOI:** 10.3389/fmicb.2020.589640

**Published:** 2020-10-15

**Authors:** Lantian Zhou, Yue Zhang, Yongze Ge, Xuan Zhu, Jianyi Pan

**Affiliations:** Zhejiang Provincial Key Laboratory of Silkworm Bioreactor and Biomedicine, College of Life Sciences and Medicine, Zhejiang Sci-Tech University, Hangzhou, China

**Keywords:** biofilm formation, quorum sensing, QS inhibitors, quorum quenching, bacteria

## Abstract

A biofilm is an assemblage of microbial cells attached to a surface and encapsulated in an extracellular polymeric substance (EPS) matrix. The formation of a biofilm is one of the important mechanisms of bacterial resistance, which not only leads to hard-to-control bacterial infections in humans and animals but also enables bacteria to be a major problem in various fields, such as food processing, wastewater treatment and metalworking. Quorum sensing (QS) is a bacterial cell-to-cell communication process that depends on the bacterial population density and is mediated by small diffusible signaling molecules called autoinducers (AIs). Bacteria use QS to regulate diverse arrays of functions, including virulence and biofilm formation. Therefore, the interference with QS by using QS inhibiting agents, including QS inhibitors (QSIs) and quorum quenching (QQ) enzymes, to reduce or even completely repress the biofilm formation of pathogenic bacteria appears to be a promising approach to control bacterial infections. In this review, we summarize the mechanisms of QS-regulating biofilm formation and QS-inhibiting agents that control bacterial biofilm formation, strategies for the discovery of new QS inhibiting agents, and the current applications of QS-inhibiting agents in several fields to provide insight into the development of effective drugs to control pathogenic bacteria.

## Introduction

A biofilm is a large number of bacterial cell aggregates coated in an extracellular mucous comprised of a polysaccharide matrix, lipids, and proteins, which they secrete (Sutherland, 2001; Branda et al., 2005). Bacteria tend to form biofilms when exposed to external environmental pressure, such as extreme nutrient deficiency or excess, high osmotic pressure, low pH, oxidative stress, antibiotics and antimicrobial agents (Costerton et al., 1994). It is a state of self-protection formed when bacteria grow on the surfaces of objects under natural conditions. Any bacteria in nature can form a biofilm under mature conditions, and more than 90% of bacteria live and grow primarily in the form of biofilms (Donlan, 2002).

Biofilms may irreversibly form on a wide variety of surfaces, including living tissues, industrial or potable water system piping, indwelling medical devices, and natural aquatic systems, and once it is formed, it cannot be removed by gentle rinsing (Donlan, 2002). More than 60% of all bacterial infections are caused by biofilm formation, according to a public announcement from the National Institutes of Health (NIH) (Lewis, 2001). The formation of biofilms leads to not only common bacterial infections, such as infections of the urinary tract, catheters, children’s middle-ear, common dental plaque formation, and gingivitis, but also to hard-to-treat or relapsing infections and severe infections that cause serious morbidity and mortality (Lewis, 2001).

Currently, the use of antibiotics is still the major treatment for bacterial infectious diseases. However, biofilms, being a barrier that exists around bacterial cells, reduces the susceptibility of bacteria to antibiotics and causes persistent infections (Stewart, 2002). It has been shown that bacteria in a biofilm increase their resistance against antibiotics by about 1000-fold (Hoiby et al., 2010). Thus, it is hard to control bacterial infections with conventional antibiotics due to the presence of biofilms. Therefore, it is urgent to find a strategy to inhibit the formation of biofilms to control these increasingly serious infections.

At present, it is well known that bacteria forming a biofilm is under the control of the quorum sensing system (QS) (Ding et al., 2011). The QS involves cell-to-cell communication among bacteria using small diffusible chemical signaling molecules called autoinducers (AIs) (Waters and Bassler, 2005). The signaling molecules accumulate in the surrounding environment with an increase of bacterial density. When the concentration of signaling molecules reaches a minimal threshold, they bind to receptor proteins, thereby activating the expression of genes associated with biofilm formation (Williams, 2007). QS inhibiting agents, including QS inhibitors (QSIs) and quorum quenching (QQ) enzymes, can cut off QS cell communication via a variety of mechanisms, consequently inhibiting the formation of biofilms (Augustine et al., 2010; Chatterjee et al., 2017). In addition, QS inhibiting agents can also increase bacterial sensitivity to antibiotics (Ozcan et al., 2019). Therefore, the use of QS inhibiting agents would be a promising approach to control bacterial infections.

In this review, we summarize the mechanisms by which QS inhibiting agents regulate biofilm formation, some strategies for the discovery of new QS inhibiting agents, and the current applications of QS inhibiting agents in medical and industrial fields. We aim to provide a new perspective for exploring more effective antibiotic drugs through the use of QS inhibiting agents.

## Mechanisms of Biofilm Formation Regulated by QS

Quorum sensing regulatory networks are not only very complicated but also vary among bacterial species (Solano et al., 2014). Therefore, the regulatory mechanisms of QS on biofilm formation cannot be described in general. However, based on the types of employed AIs, QS systems can be divided into several categories, namely, AHL system, AIP system (these two system known as AI-1 system previously), AI-2 system and AI-3 system.

The AHL system exists in Gram-negative bacteria and the signaling molecules employed in this system are *N*-acyl homoserine lactones (AHLs) (Slock et al., 1990; Stevens et al., 1994). While, autoinducing peptides (AIPs) are employed in the AIP system and this system is found only in Gram-positive bacteria (Kleerebezem et al., 1997; Irie and Parsek, 2008). Presently, there are a large number of studies on these two systems.

However, there are only a few reports about AI-2 and AI-3 systems, although these two systems have been found to be present in both Gram-positive and Gram-negative bacterial species and to participate in interspecies signal exchange (Surette and Bassler, 1998). AI-2 signaling molecules are a class of furanosyl borate diesters whose precursors are 4,5-dihydroxy-2,3-glutara dione (DPD) (Bassler et al., 1997). The AI-3 signaling molecules have recently been identified as pyrethroids (Sperandio et al., 2003; Kim et al., 2020). The mechanism of how the QS system regulates biofilm formation is described in detail below for Gram-negative and positive bacteria separately.

### QS Regulation of Biofilm Formation in Gram-Negative Bacteria

The signaling molecule of the AHL system in Gram-negative bacteria is AHL. The AHL-mediated QS system was first found in *Vibrio fischeri* (Nealson and Hastings, 1979). In this bacterium, the AHL signal, *N*-(3-oxohexanoyl)-L-homoserine lactone (OHHL), is biosynthesized by AI synthase LuxI, and the resulting OHHL diffuses out of the bacterial cell. When the concentration of OHHL reaches a critical threshold with the increase of cell density, OHHL binds to LuxR, which is not only an OHHL receptor, but also a DNA-binding transcriptional activator, thereby activating the expression of genes associated with biofilm formation (Engebrecht et al., 1983; Engebrecht and Silverman, 1984). Presently, it is well known that this regulatory process is a typical model for the regulation of biofilm formation by AHL systems in most Gram-negative bacteria.

Take *Pseudomonas aeruginosa* for instance, which has two AI synthase genes, *lasI* and *rhlI*, which both share significant sequence homologies to *luxI* of *V. fischeri* (Lee and Zhang, 2015). Their signals, *N*-(3-oxo-dodecanoyl)-l-homoserine lactone (OdDHL) and *N*-butyryl-l-homoserine lactone (BHL), are separately synthesized by LasI and RhlI. When they reach the concentration threshold, these two AHL signaling molecules bind to their receptors, LasR and RhlR, respectively, to activate the expression of regulatory genes related to biofilm formation and virulence (Wade et al., 2005). Among these two AHL systems, the *rhl* system is involved in regulating swarming motility that participates in the early stage of biofilm establishment (Khan et al., 2020c), and the biosynthesis of virulence factors, such as rhamnolipid and pyocyanine (Winzer et al., 2000; Daniels et al., 2004; Dusane et al., 2010). The *las* system controls genes encoding elastase, alkaline protease, endotoxin A and other genes related to biofilm formation (Wilder et al., 2011).

In addition, in *P. aeruginosa*, there are also two other types of AHLs-mediated systems, the *pqs* system and the *iqs* system. These two systems work in a way similar to the *rhl* and *las* systems, though their AIs, PQS (2-heptyl3-hydroxy-4-quinolone) and IQS (2-(2-hydroxyphenyl)-thiazole-4-carbaldehyde), are chemically different from AHLs (Lee and Zhang, 2015). Additionally, the *pqs* system has been reported to be related to the synthesis of bacterial extracellular DNA, which is important for the formation of biofilms (Allesen-Holm et al., 2006). In brief, the four AHL systems, *las*, *rhl*, *iqs* and *pqs*, cross-interact to form a complicated QS network that co-regulates biofilm formation by *P. aeruginosa*.

The AHL system is also involved in the regulation of biofilm formation in *Escherichia coli* (Walters and Sperandio, 2006). However, different from the AHL system in *P. aeruginosa*, only the receptor gene *sdiA* homologous to *luxR* is found and the AHL synthase gene homologous to *luxI* is absent (Walters and Sperandio, 2006). Hence, it is speculated that the receptor SdiA may respond to the AHLs produced by other bacterial species to regulate biofilm-related gene expression. The finding that in the presence of exogenous AHLs, there is an increase of EPS production in *E. coli* and the attachment of bacterial cells (Aswathanarayan and Vittal, 2016) confirms that bacteria can utilize the signaling molecules of other bacterial species for biofilm formation.

Currently, AI-2 systems have been found to affect biofilm formation in several Gram-negative species, such as *Helicobacter pylori*, *E. coli, V. parahemolyticus*, *P. aeruginosa* and *V. cholerae* (Hammer and Bassler, 2003; Li et al., 2007; Anderson et al., 2015; Li et al., 2015; Guo et al., 2018). However, at present, only the regulatory mechanisms of the AI-2 systems in *E. coli* and *V. cholerae* have been clarified. In *E. coli*, the AI-2 signaling molecule is transported into the cell by an ABC transporter protein when the extracellular concentration of the AI-2 signaling molecule reaches its threshold (Li et al., 2007). The resulting signaling molecule is then phosphorylated by LsrK kinase followed by binding to the transcriptional regulator LsrR, thereby activating gene expression (Li et al., 2007). In *V. cholerae*, the AI-2 signaling molecule is detected by the receptor complex LuxPQ. When the signaling molecule concentration reaches its threshold, the kinase activity of LuxQ is converted to phosphatase, which dephosphorylates downstream regulatory protein LuxO, resulting in the production of transcriptional regulatory protein HapR, thereby inhibiting the transcription of genes related to biofilm formation (Hammer and Bassler, 2003, 2007).

AI-3 signaling molecules are currently known to be related to the formation of flagellum and adhesin in *E. coli* (Sperandio et al., 2002), but their regulatory mechanisms for biofilm formation remain unclear.

### QS Regulation of Biofilm Formation in Gram-Positive Bacteria

In Gram-positive bacteria, the AIs of the AIP system are AIPs, and the regulation of biofilm formation by the AIPs-mediated QS system is also a typical pattern. Bacteria produce a small oligopeptide in their cells, and the oligopeptide is processed into a mature AIP through modification and then it is transported outside of the cells (Sturme et al., 2002). When the concentration of AIP reaches its threshold, it binds to the extracellular segment of histidine kinase, a transmembrane receptor localized on the cell membrane, which leads to the activation of the kinase, followed by phosphorylation of downstream response regulatory factors, resulting in regulation of the expression of genes related to biofilm formation (Sturme et al., 2002).

For example, in *Staphylococcus aureus*, five genes, *agrA*, *agrB*, *agrC*, *agrD* and *hld* in the *agr* operon constitute the *agr* AIP system (Painter et al., 2014). The signaling AIP is converted from its precursor peptide AgrD, and AgrB, a transmembrane protein, is responsible for the conversion of AgrD to mature AIP and transportation of the resulting AIP outside of the cell. When the extracellular AIP concentration reaches its threshold, the AIP binds to an extracellular part of AgrC, an integral transmembrane protein functioning as a histidine kinase, resulting in the activation of the kinase. The activated AgrC in turn phosphorylates the downstream response regulator AgrA. Phosphorylated AgrA binds to the intergenic DNA between promoters P2 and P3, and thus activates promoter transcription. The *agr* system has been shown to play a major role in the dispersion phase, which is the last stage of biofilm formation (Yarwood et al., 2004). *Agr* mutants formed a thicker biofilm comparing with the wild type, but this increased biofilm thickness has been attributed to the inability of cells to detach from the mature biofilm, not to cell growth or death (Vuong et al., 2000, 2004).

Presently, there are several reports about the regulation of biofilm formation by the AI-2 system in Gram-positive bacteria. For example, a study has shown that the lack of *luxS*, which encodes AI-2 synthase, promotes the transcription of *rbf*, a positive regulatory factor for biofilm formation, and results in an increase of biofilm formation and higher levels of production of polysaccharide intercellular adhesion (PIA) in *S. aureus* (Ma et al., 2017). However, the opposite finding, that the absence of *luxS* decreases biofilm formation, was also found in *Enterococcus faecalis* (Wang et al., 2011; Yang et al., 2018) and *Streptococcus suis* (Wang et al., 2011; Yang et al., 2018). Nevertheless, the AI-2 system is involved in the regulation of biofilm formation in Gram-positive bacteria, but its regulatory mechanism has not yet been fully characterized.

In addition, there is still no report about the AI-3 system in regard to the control of biofilm formation in Gram-positive bacteria.

## The Mechanisms of QS Inhibiting Agents Suppressing Biofilm Formation

**FIGURE 1:**
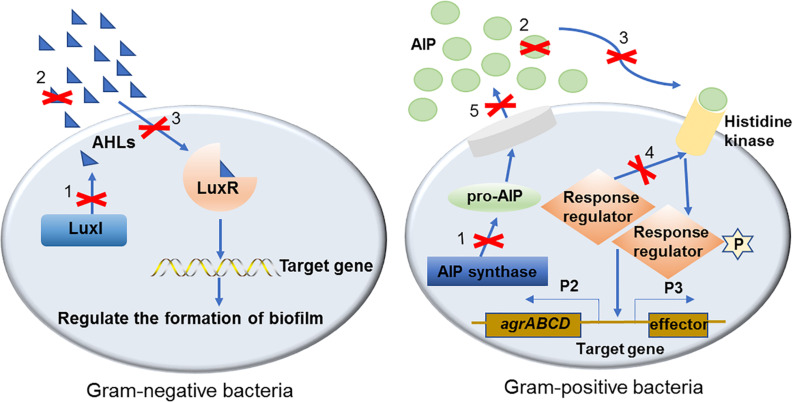
The mechanisms of QS inhibiting agents in controlling bacterial biofilm formation. Mechanisms of QS inhibiting agents in controlling bacterial biofilm formation are marked with numbers on the diagram: (1). Inhibit AIs synthesis; (2). Degrade or inactivate AIs by AHL-lactonases, oxidoreductases, antibodies, etc.; (3). Interfere with the signal receptors using AI antagonists; (4). Interfere with the response regulators thus disturbing signaling cascade; (5). Reduce the extracellular AIs accumulation by inhibiting AIs efflux hence inhibited cell-to-cell signaling.

In the past two decades, a number of effective QS inhibiting agents have been developed and successfully used to control the formation of bacterial biofilms. These QS inhibiting agents are mainly QS inhibitors (QSIs) and quorum quenching (QQ) enzymes. Although different types of QSIs or QQ enzymes interfere with different parts of the QS signaling pathway, the inhibitory mechanism can be divided into three categories based on their functional targets, i.e., targeted to AI signaling molecules, receptors and downstream signaling cascades (Figure 1). These three mechanisms are described in detail with examples as follows.

### Target Signal Molecule

The QS inhibiting agents that target the AI signaling molecules are mainly AHL-lactonases, oxidoreductases, antibodies, and some other molecular compounds (Table 1). These agents inhibit the QS system by inactivation of signaling molecule synthases, neutralization of AIPs with antibodies, modification or degradation of the signaling molecules, etc.

**TABLE 1:** Studies on controlling biofilm by targeting QS signaling molecule.

Source	QS-inhibiting agents	Chemical structure	Target bacteria	Effects	References
*Bacillus cereus* VT96	AHL-lactonase AiiA	NA	*P. aeruginosa* *V. cholerae* *E. cloacae*	Degraded AHLs, prevent the biofilm formation and production of virulence factors	Augustine et al., 2010; dos Reis Ponce et al., 2012; Anandan and Vittal, 2019
Synthesis	Molecularly imprinted polymers (MIPs)	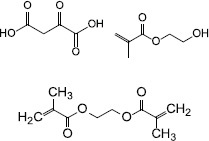	*P. aeruginosa*	Captured OdDHL, therefor interrupted QS, and subsequently inhibit biofilm formation	Ma et al., 2018
*Arthrobacter nitroguajacolicus* strain Rü61a	3-Hydroxy-2-methyl-4(1H)-quinolone 2,4-dioxygenase Hod	NA	*P. aeruginosa*	Catalyzed the conversion of PQS to N-octanoylanthranilic acid and carbon monoxide, reduced the expression of the PQS-regulated virulence	Pustelny et al., 2009
Derivative	Boronic acid derivate SM23	NA	*P. aeruginosa*	Decreased 3-oxo-C12-HSL and C4-HSL and reduced biofilm formation	Peppoloni et al., 2020
Synthesis	Acyl-HSL analog J8-C8	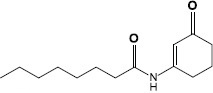	*B. gluma*	Bound to TofI, disturbed C8-HSL synthesis, affected biofilm formation	Chung et al., 2011
Synthesis	Diketopiperazines	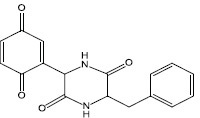	*B. cenocepacia*	Interfered with the activity of signal molecule synthase CepI and rendered the bacteria unable to produce biofilm	Buroni et al., 2018
Synthesis	Anti-autoinducer monoclonal antibody AP4-24H11	NA	*S. aureus* RN4850	Sequestrated the autoinducing peptide (AIP)-4, inhibited QS and biofilm formation	Park et al., 2007
Gene from a soil metagenome	NADP-dependent reductase BpiB09	NA	*P. aeruginosa* PAO1	Reduced pyocyanin production, decreased motility, poor biofilm formation	Bijtenhoorn et al., 2011
Synthesis	3-(dibromomethylene) isobenzofuran-1(3H)-one derivatives	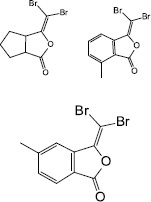	*F. nucleatum* *P.gingivalis* *T. forsythia*	Inhibited biofilm formation through the inhibition of AI- 2 activity	Park et al., 2017

*NA, not available.*

Degradation or neutralization of QS signaling molecules by QQ enzymes are a direct and effective way to inhibit the QS system. In Gram-negative bacteria, AHLs can be degraded by two types of hydrolases, AHL-lactonase and AHL-acylase. An AHL-lactonase, encoded by *aiiA* of *Bacillus spp* and belonging to the metallo-β-lactamase superfamily, has been determined to effectively inhibit biofilm formation and attenuate virulence factors in several bacterial species (Augustine et al., 2010; dos Reis Ponce et al., 2012; Anandan and Vittal, 2019). Another QQ enzyme, AHL-acylase, is not widespread in Gram-negative bacteria. However, the enzyme has been found to be present at least in *P. aeruginosa*, and it has been shown to degrade AHLs with side chains and hence enable the bacteria to modulate the QS system (Sio et al., 2006). Currently, there are few reports on the degradation of AIPs by QQ enzymes in Gram-positive bacteria. However, AIPs can be neutralized by antibodies and cause interruption of QS signaling. For example, an anti-AI monoclonal antibody can efficiently inhibit QS via neutralization of an AI peptide (AIP-4) that is produced by *S. aureus* (Park et al., 2007).

*N*-acyl homoserine lactones oxidoreductase, another class of QQ enzyme, has also been reported in Gram-negative bacteria, it modifies the AIs and thereby attenuates the specific binding of the AIs to the corresponding receptors, resulting in a decrease of biofilm formation. BpiB09 is a metagenome-derived NADP-dependent reductase and it has been found to be involved in inactivation of the QS signaling molecule OdDHL. Although the AHLs of *P. aeruginosa* are probably not the native substrate of BpiB09, the expression of the enzyme in *P. aeruginosa* results in poor biofilm formation, significantly reduced pyocyanin production and decreased motility (Bijtenhoorn et al., 2011). Another AHL oxidoreductase, Hod, a 2,4-dioxygenase, is capable of catalyzing the conversion of PQS to *N*-octanoylanthranilic acid and carbon monoxide. Exogenous supplementation of Hod protein into *P. aeruginosa* cultures reduces expression of the PQS biosynthetic enzyme PqsA and the PQS-regulated virulence factors (Pustelny et al., 2009). In short, these QQ enzymes can effectively inhibit bacterial biofilm formation.

Inhibiting the biosynthesis of QS signaling molecules may be a more direct way to interrupt the QS system and inhibit biofilm formation. Some QS inhibitors have been found to inhibit the synthetic activities of AIs synthases. TofI is an AHL synthase identified in the Gram-negative bacterium *Burkholderia gluma*. The binding site of TofI can be occupied by an AHL analog and therefore disturb AHL synthesis (Chung et al., 2011). Another example is diketopiperazines that target CepI, an AHL synthase of *B. cenocepacia*, can interfere with the activity of signaling molecule synthases, rendering the bacteria unable to produce biofilms (Buroni et al., 2018).

### Targeting the Signaling Molecule Receptors

The second mechanism of QS inhibiting agents is by targeting the receptors of the QS signaling molecules, thereby inactivating the receptor or competing for the receptor. In most cases, the ligand binding domains of receptors with the native AIs are highly conserved (Khan et al., 2019), which can be competitively or non-competitively bound by most AIs (Table 2). Two classes of QS inhibiting agents, flavonoids and furanones, have been found that can bind to receptors of a variety of pathogenic bacteria (Paczkowski et al., 2017; Proctor et al., 2020). A plant flavonoid, naringenin, competes with the physiological signaling molecule OdDHL by directly binding to the receptor LasR, resulting in inhibition of the production of the QS-regulated virulence factors, pyocyanin and elastase, etc., in *P. aeruginosa* (Hernando-Amado et al., 2020). Also in *P. aeruginosa*, the receptor LasR can interact with sitagliptin, a drug used for the treatment of diabetes mellitus type 2, and a minor inhibitory concentration of sitagliptin significantly inhibits biofilm formation (Abbas et al., 2020). In addition, flavonoids can non-competitively bind to the LasR LBD and prevent the protein from binding to DNA, causing repression of certain QS behaviors (Paczkowski et al., 2017). Moreover, some QS inhibiting agents can bind to different receptors at the same time. For example, 3-benzene lactic acid (PLA), a QS inhibiting agent produced by Lactobacillus, antagonistically binds to the receptors RhlR and PqsR with a higher affinity than its cognate ligands BHL and PQS in *P. aeruginosa* (Chatterjee et al., 2017).

**TABLE 2:** Studies on controlling biofilm by targeting QS signaling receptors.

Source	QS-inhibiting agents	Chemical structure	Target bacteria	Effects	References
Lactobacillus	3-Phenyllactic acid (PLA)	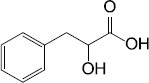	*P. aeruginosa*	Bound to QS receptors RhlR and PqsR with high affinity, thus inhibited the expression of virulence factors such as protease, pyocyanin and rhamnolipids that are involved in the biofilm formation	Chatterjee et al., 2017
Synthesis	Sitagliptin	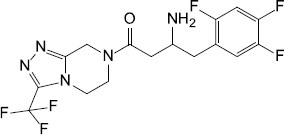	*P. aeruginosa*	Interacted with LasR receptors, and significantly inhibited the biofilm formation	Abbas et al., 2020
Plant	Naringenin	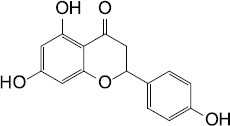	*P. aeruginosa*	Competed with OdDHL by directly binding the QS regulator LasR, inhibited the production of the QS-regulated virulence factors, pyocyanin and elastase	Hernando-Amado et al., 2020
Plant	Fructose-furoic acid	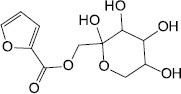	Uropathogenic *E. coli*	Competed with the SdiA native ligand C8HSL to down regulate its target specific expression and biofilm phenotypic characters	Vinothkannan et al., 2018
Synthesis	Furanones	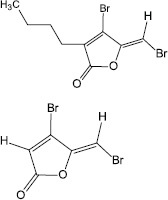	*P. aeruginosa*	Competed with the native autoinducers to bind to the AHL receptors, and significantly decreased virulence factor production and biofilm formation	Proctor et al., 2020
Synthesis	Meta-bromo-thiolactone	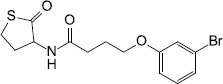	*P. aeruginosa*	Inhibited receptors LasR and RhlR, prevented virulence factor expression and biofilm formation	O’Loughlin et al., 2013
Synthesis	N-phenyl-4-(3-phenylthioureido) benzenesulfonamide	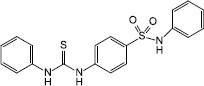	*E. coli*(EAEC) O104:H4	allosterically modified AI-3 receptor QseC, impeded virulence expression and decreased biofilm formation	Curtis et al., 2014

As another class of QS inhibiting agents, furanones, can compete with the native AIs to bind to, and subsequently block, the AHL receptors. They have been demonstrated to significantly decrease virulence factor production and biofilm formation in a range of bacterial species (Proctor et al., 2020). In addition to competing with signaling molecules for receptors, some QS inhibiting agents, such as meta-bromo-thiolactone, can also directly inactivate the receptors to prevent virulence factors expression and biofilm formation (Sully et al., 2014).

### Blocking the Signaling Cascade

The third mechanism of inactivation of QS systems is blocking the signaling cascade by deactivating the downstream response regulators or other regulatory factors. For example, in the AIP system of *S. aureus*, downstream response regulator AgrA is phosphorylated and thereby activated, which is triggered by upstream signaling, and it binds to DNA sequences associated with promoters and upregulates the expression of relevant genes as described above. Inhibiting the response regulators can block the signaling cascade and prevent the formation of a bacterial biofilm (Table 3). Savarin, for example, is a small molecule identified as an *S. aureus* virulence inhibitor, which can specially target AgrA to disrupt *agr* operon-mediated QS, and hence inhibit biofilm formation (Sully et al., 2014).

**TABLE 3:** Studies on controlling biofilm by blocking the signaling cascade.

Source	QS-inhibiting agents	Chemical structure	Target bacteria	Effects	Reference
Synthesis	Savirin	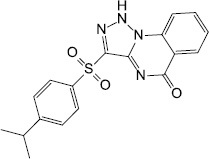	*S. aureus*	Targeted AgrA to disrupt *agr* operon-mediated QS	Sully et al., 2014
Synthesis	Virstatin	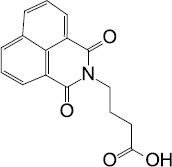	*A. nosocomialis*	Repressed the expression of AnoR, leading to decreased synthesis of OH-dDHL, thus adversely affecting the signal transduction cascade, reducing biofilm formation and motility	Oh and Choi, 2015
Synthesis	Curcumin	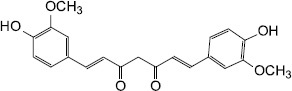	*P. aeruginosa* PAO1	Inhibited QS controlled protease and biofilm formation	Sethupathy et al., 2016
Synthesis	Efflux pumps inhibitor PAβN	NA	*P. aeruginosa* clinical isolate	Reduced the extracellular accumulation of QS signals and significantly diminished the relative expression of QS cascade (*pqsA, pqsR, lasI, lasR, rhlI* and *rhlR*)	El-Shaer et al., 2016

*NA, not available.*

In addition to acting on the response regulator, the QS inhibiting agents can also act on other regulatory factors to block signaling cascades. For example, virstatin, a small molecule that acts to prevent the expression of cholerae virulence factors, can repress the expression of AnoR, which is a positive regulator of the LuxI-like synthase AnoI in *Acinetobacter nosocomialis*, leading to decreased synthesis of *N*-(3-hydroxy-dodecanoyl)-L-homoserine lactone (OH-dDHL), thus affecting the signaling cascade, and reducing biofilm formation and motility (Oh and Choi, 2015). Moreover, efflux pumps inhibitor PAβN reduces the extracellular accumulation of QS signaling molecules and significantly decreases the relative expression of the QS cascade (*pqsA*, *pqsR*, *lasI*, *lasR*, *rhlI* and *rhlR*) in *P. aeruginosa* clinical isolates, and this is followed by a reduction of bacterial virulence (El-Shaer et al., 2016).

## Strategies of Exploiting New QS Inhibiting Agents

In the past two decades, researchers have discovered plenty of QS inhibiting agents that can effectively inhibit biofilm formation in bacteria. The development of new QS inhibiting agents that can effectively replace antibiotics has been a hot topic in the antibacterial research field. With the increasing degree of bacterial resistance to antibiotics, it has become more urgent to develop new QS inhibiting agents that can effectively inhibit biofilm formation. Here, we will introduce four strategies for exploiting new QS inhibitors.

### Synthesis of Derivatives of Known QSIs

Some QS inhibitors are usually structural analogs of AIs, which cause the inactivation of QS by competitively binding to the receptor. Therefore, the synthesis of derivatives of known QSIs that do not alter the core structure may be an effective strategy. Actually, researchers have used natural and chemically synthesized halogenated furanones to successfully synthesize furanone analogs bearing alkyl chains, vinyl bromide keys or aromatic rings, and these analogs can also inhibit biofilm formation (Chang et al., 2019). In addition, C-5 aromatic substituted furanones have been designed and synthesized based on 5-hydroxyl-3,4-halogenated-5H-furan-2-ones, and this compound shows remarkable inhibition of biofilm formation as well as inhibition of virulence factor production in *P. aeruginosa* (Chang et al., 2019). Moreover, a new class of brominated furanones that contained a bicyclic structure were designed and synthesized, and this class of molecules exhibited reduction in the toxicity to mammalian cells, but retained the inhibitory activity toward biofilm formation of bacteria (Yang et al., 2014).

### Modification of Existing QQ Enzymes

The use of quenching enzymes to interfere with QS is an attractive strategy to fight bacterial infections. Intentional modification of QQ enzymes by using protein engineering methods may obtain greater efficiency and stability of quenching enzymes. Thus, this could be an effective strategy for developing new QQ enzymes. MomL is a marine lactonase that can degrade various AHLs. Two MomL mutants, MomL^I144V^ and MomL^V149A^, exhibit higher activities to block the production of virulence factors of *Pectobacterium carotovorum subsp*. *carotovorum* (Pcc) (Wang et al., 2019). PvdQ is an acylase with effective QQ activity in *P. aeruginosa*, but it hardly hydrolyses short-chain AHLs. However, its variant PvdQ^Lα^ ^146W,Fβ^ ^24*Y*^ has an altered substrate specificity, and it exhibits high hydrolysis activity toward shorter-chain AHLs such as C8-HSL (Koch et al., 2014).

### Search for QS Inhibiting Agents in Natural Products

There are abundant biochemical resources in plants and microorganisms in nature. In recent years, a number of natural products have been found to have potential abilities of inhibition of QS signaling and biofilm formation with the extensive use of computer-aided programs like structure-based virtual screening (SB-VS) and molecular docking bioassays (Ahmed et al., 2019; Borges and Simoes, 2019). Natural plant-derived compounds trans-cinnamaldehyde (CA) and salicylic acid (SA) can significantly inhibit the expression of QS-regulated genes involved in virulence, rhamnolipid and reduced biofilm formation in *P. aeruginosa* (Ahmed et al., 2019). Recently, a novel AHL acylase, MacQ, has been identified from a multidrug-resistant bacterium, *Acidovorax sp.* strain MR-S7, and it was able to degrade a wide variety of AHLs, ranging from C6 to C14 side chains with or without 3-oxo substitutions, thus interfering with the QS system in the bacterial pathogen (Kusada et al., 2017).

### Identify Approved Drugs as QS Inhibiting Agents

The search for QS inhibiting agents from the library of drugs approved for clinical applications may be a promising way to shorten the period of development of an anti-QS drug from discovery to clinical use, since long-term clinical trials are needed to ensure QS inhibiting agents are safe and reliable before they can be used to treat infectious diseases. A typical example of a new therapeutic use of an old drug is niclosamide, which is an FDA approved anti-helminthic drug. Niclosamide has been identified to strongly inhibit the QS response by targeting the *las* QS system, thereby reducing the expression of *las* regulon-controlled virulence factors and biofilm formation in *P. aeruginosa* (Imperi et al., 2013). Another FDA approved drug that is used for the treatment of upper respiratory tract infections and for tracheobronchial infections caused by Gram-positive pathogens, clofoctol, has also been found to significantly reduce biofilm formation through inhibiting the *pqs* QS system in *P. aeruginosa* (D’Angelo et al., 2018). In addition, albendazole, also an FDA approved clinical drug, has been found to have great potential to act as a QS inhibitor. It quenches the QS by interacting with the hydrophobic amino acid residues of the hydrophobic pocket of CviR and LasR receptors in *P. aeruginosa* (Singh and Bhatia, 2018). Antibiotics have also shown great potential as QS inhibiting agents. For exsample, aminoglycosides, a commonly used class of antibiotics, exhibit biofilm inhibition by targeting the QS regulatory protein LasR in *P. aeruginosa* (Khan et al., 2020a; Khan et al., 2020b).

## Applications of QS-Inhibiting Agents in Controlling Bacterial Biofilm Formation

As a new type of antimicrobial agent, QS inhibiting agents have been applied in several fields, such as in medical treatments, food processing and water treatment.

Currently, QS inhibiting agents are widely used in health-related fields. *P. aeruginosa* is a major cause of nosocomial infections, especially in the pulmonary infections associated with cystic fibrosis. This organism shows a remarkable capacity to resist antibiotics. Alternative drugs have proven useful against this multiresistant strain. The acylase PvdQ has been identified as an QS inhibiting agent that can irreversibly hydrolyze AHL signaling molecules and has great therapeutic efficacy against pulmonary infections in a mouse model (Utari et al., 2018).

In addition, owing to biofilms being one of the important virulence factors of pathogenic bacteria, infections resulting from the formation of biofilms on medical devices remains a significant clinical problem (Ozcelik et al., 2017). Therefore, a poly(ethylene glycol) (PEG) based multifunctional coating that allows for the covalent incorporation of the synthetic QS inhibitor 5-methylene-1-(prop-2-enoyl)-4-(2-fluorophenyl)-dihydropyrrol-2-one (DHP) in a surface can reduce biofilm formation, and the use of this coating can reduce bacterial colonization (Ozcelik et al., 2017). Obviously, this provides a useful way to prevent device-related infections. Moreover, QS inhibiting agents can also be used as antibiotic accelerants for treating bacterial infections. Two cinnamic acid derivatives, 4-dimethylaminocinnamic acid (DCA) and 4-methoxycinnamic acid (MCA) as AHL inhibitors, were both found to not only markedly inhibit biofilm formation, but also to enhance the susceptibility of biofilms to tobramycin (Cheng et al., 2020).

In addition to great application prospects in the health-related fields, QS inhibiting agents can also be used in the food industry. Food safety has always been a major concern in the food industry. The formation of biofilms enhances the attachment of bacterial pathogen to the surface of food packaging bags or processing equipment, which increases post-processing contamination and risks to public health due to their persistence and resistance to cleaning and disinfection procedures (Shi and Zhu, 2009). Thus, the extraction of QS inhibiting agents from foodborne materials to solve food contamination problems is a relatively effective approach. The essential oils that were extracted from *Murraya koenigii* were found to have strong QS inhibitory and anti-biofilm activities that can reduce cell attachment, metabolic activity and EPS production, and in addition, they could delay the decomposition of refrigerated milk caused by *psychrophila* PSPF19 (Bai and Vittal, 2014). Additional applications of QS inhibiting agents in the food industry are still being explored.

At present, the application of quenching QS in water treatment has also been reported, and it shows great potential and commercial prospects. In wastewater treatment, membrane bioreactor (MBR) is an important technique that combines the activated sludge and membrane filtration processes (Yeon et al., 2009a, b; Lee et al., 2018; Oh and Lee, 2018). However, biofouling, which is mainly caused by the generation of a thickened biofilm layer resulting from bacterial gathering, is a primary problem during the use of MBR (Kose-Mutlu et al., 2019), which seriously affects the efficiency of wastewater treatment. QQ enzymes can be employed to prevent biofouling in MBR. For instance, Kim et al. (2011) immobilized a QQ enzyme (acylase) onto a nano-filtration membrane, and found the acylase-immobilized membrane prohibits the formation of the mushroom-shaped mature biofilm and prevents more than 90% of the initial flux after 38 h operation, while the un-immobilized raw membrane dropped to 60% accompanied by severe biofouling.

There are studies showing that the use of QS inhibiting agents may also be an effective strategy to eradicate biofilm contamination in metalworking fluids (MWFs). Biofilm contamination is a critical problem in MWFs, which not only affects product quality, but also shortens the lifetime of MWFs. Two QS inhibiting agents, patulin and furanone C-30, both reduced biofilm formation in MWF when compared to untreated controls (Ozcan et al., 2019).

## Conclusion and Future Research

Biofilms can promote bacteria survival in harsh environments. Conventional antibiotics and bactericides cannot penetrate the extracellular matrix of biofilms, resulting in decreased bacterial sensitivity, and therefore, biofilm-related pollution poses serious problems in many fields, including the environment, food, and human diseases. The formation of a biofilm is regulated by the QS system, and thus the use of QS inhibiting agents is a promising strategy to control biofilm formation, and it has been successfully applied in a number of fields.

QS inhibiting agents control biofilm formation generally by targeting QS signaling molecules or their receptors, or downstream regulatory factors. They do not kill bacteria or inhibit the growth of bacteria, and instead they interfere with the expression of virulence factors and inhibit biofilm formation, which puts less pressure on bacterial survival and reduces their drug resistance. This is completely different from the mechanism of antibiotics killing bacteria.

In recent years, many natural or synthetic QS inhibiting agents that effectively reduce biofilm formation have been exploited. However, there are still several problems to be overcome: (1) the mechanisms of some QS inhibiting agents in controlling biofilms are still unclear; (2) some known QS inhibiting agents are cytotoxic or unstable; (3) the anti-biofilm effect is not broad enough, only being effective against one or two bacterial species; (4) what are the best conditions for QS inhibiting agents in controlling biofilms? (5) whether QS inhibiting agents have an impact on beneficial bacteria in the environment. These problems all need to be overcome to develop more efficient, less toxic QS inhibiting agents that will provide great value in the future for biofilm prevention and treatment.

## Author Contributions

JP and LZ provided the general concept and designed the manuscript and revised and approved the manuscript. LZ, YG, YZ, and XZ wrote the manuscript. LZ contributed to create the figure. All authors contributed to the article and approved the submitted version.

## Conflict of Interest

The authors declare that the research was conducted in the absence of any commercial or financial relationships that could be construed as a potential conflict of interest.
